# Sick Nursing in the Sixteenth Century

**Published:** 1887-10-29

**Authors:** 


### Sick Nursing in the Sixteenth Century.

By a Student of Mediæval Institutions.

(*Concluded from p. 16.*)

The code of directions (briefly known as "the Rule") in accordance with which the sisterhood of St. Vincent de Paul is governed bears eloquent testimony to the elevation of S. Vincent's character as well as to the sagacity of his judgment.

Chapter I speaks of the great aim of the congregation as a whole and of the individual sisters. They are called and assembled "to honour our Lord Jesus Christ as the source and model of all charity, serving Him corporally and spiritually in the person of the poor, whether sick or infants or prisoners," and for this end "they will unite the interior exercises of the spiritual life to exterior works (*emplois*) of Christian charity towards the poor, conforming to these rules . . . . as the best means of arriving at this end." Having practical every-day work to perform, they are, as compared with the members of other orders, at some disadvantage, "having for monasteries only the houses of the sick, for cell only a hired room, for their chapel only the parish church, for cloisters only the streets of the city or the wards of a hospital; for enclosure, obedience; for the grille, the fear of God; and for veil, holy modesty." They are to despise self and the things and interests of earth, preferring "lowly services and those repugnant to natural inclination to those which are honourable and pleasant." They are to detach themselves from all earthly affections, whether for things, places, or persons, even their parents or confessors; they are to be ready to leave all at the call of duty, "suffering bravely (*de bon cœur*) and for the love of God, inconveniences, mockeries, calumnies, . . having a complete trust in the divine providence, and abandoning themselves thereto entirely as an infant to its nurse."

Chapter II insists upon the duty of poverty. "As the servants of the poor, they must live poorly . . . always considering that the servants of the poor ought not to be better treated than their masters." All their possessions are to be in common, "as among the primitive Christians."

Chapter III deals with the inward spirit in which their life is to be lived, and how modesty is to be cultivated. When walking abroad, and when speaking to persons of the other sex, their eyes are to be held downcast. Their movements and actions are to be dignified, and their dress and apartments neatly and cleanly kept. Article 3 of this chapter defines the discipline of the order. Obedience, submission, self-control, these are to take the place of austerities and penances. The fasts to be observed are for the most part those which any good lay Catholic would be careful to keep.

The next chapter speaks of obedience, which is to be rendered to all in due order—to the Bishop, to the Superior-General of the Lazarists, to their own Mother-General, and, when engaged in hospital work, to the officials and doctors, remembering that all human authority is a reflex of the divine. "They will be equally obedient without any delay to the sound of the bell (*au son de la cloche*), as if to the voice of our Lord, who calls them to the (spiritual) exercises of the community."

Chapters V and VI deal with the inner life of the sisters, and their relations to one another. "If it should happen, through human infirmity, that one sister has given offence to another, she shall not fail to beg pardon of her on her knees immediately, or at latest in the evening before retiring to rest, and the other shall receive humbly and in good part (*en bon cœur*) the humiliation of her sister, and shall also kneel; this holy practice being a sovereign remedy to heal quickly all bitterness of heart and resentment."

Chapter VII recurs to the practical work of the order. "Their principal employment being to serve the sick poor, they will acquit themselves therein with all the care and affection possible, remembering that it is not to the poor, but to Jesus Christ, that they render service." They are to have great regard to the spiritual condition of their patients: "they will not forget to say at some time or another a few words to dispose them to patience or to make a good general confession, to die well or to live well. They will have particular care to teach them the things necessary to salvation, and to see that they receive in good time all the sacraments." Section 8 contains the regulations for the weekly chapter. This gathering (*la petite conférence*) is to be held every Friday at half-past seven, when "each one shall confess her faults" (committed against the rules) "in the presence of the others in the accustomed manner, shall receive in good part the advice and the penance which shall be imposed, and shall ask pardon from those to whom she may have given any offence or bad example." And further, once a month each sister is to request "to be informed publicly of the faults which the others shall have remarked in her, which her associates shall do in a spirit of humility and charity," without making mention of the faults which may have been committed against themselves in particular.

Chapter IX details the manner in which the day is to be employed, and may be summarised briefly as follows. At four o'clock the Sisters of Charity rise, dress, and having made their private devotions, at half-past four join in common worship, continuing in meditation till a quarter-past five. At six o'clock they are silently to betake themselves to their household duties, assembling in the chapel at seven for mass, after which breakfast awaits them in the refectory, "where they shall take only a piece of bread," and that in silence. Breakfast over, they disperse to their avocations. At half-past eleven "they shall make *l'examen particulier*" (*i.e.*, self-examination). Dinner follows, during which proceeds *la lecture spirituelle* (*i.e.*, a reading from some pious author). Another interval of work is succeeded by an hour's recreation. At two o'clock occurs another *lecture spirituelle*, without interrupting any work that may be going forward. At three o'clock devotion again claims them, and an office of a curious character is said. "They shall kneel down, and a sister shall say aloud these words: 'Christ became for us obedient unto death, even the death of the Cross, wherefore God also hath exalted him' (Phil. ii, 8), and together they shall adore the Son of God, dying for the salvation of our souls, and offering Himself to the Eternal Father at the very moment when He gives up the ghost, praying Him to apply the merit of His death, particularly to those who are in the (last) agony, or in a state of sin, and to all the souls detained in purgatory. Having made this act for the space of three Paters and Aves, they shall kiss the earth and rise." Once more, at half-past five, work having occupied the interval, "they shall make *l'examen particulier*, and then go to supper, then to work again, with another interval of recreation. At 8.15 is the last service of the day, and by nine o'clock all lights must be out. Additional regulations respecting novices conclude the general "rule."

Then follow special directions for those sisters engaged in parish work. They are ever to bear in mind their twofold character as ministers of spiritual as well as of temporal things. They must watch each death-bed, "assisting the patients to die well, and inducing them to make short acts of devotion, praying for them and giving them holy water with an aspargil (sprinkler), pronouncing the sacred name of Jesus at the moment of death." If the patient recover, re-oubled efforts are to be made to induce him to lead a good life in the future. In all cases, however, spiritual ministrations are not to hinder temporal services, and the sisters are further "to prefer the service of the sick poor to all other exercises, whether corporal or spiritual, and are to have no scruple in expediting or delaying everything for that (work)."

Such are the rules under which the Sisters of Charity labour. That they contain a lofty ideal of the nurse's avocation, that they are instinct with the personal piety of their compiler, must be conceded by all, unable though we may be to admit the wisdom of all the methods by which S. Vincent endeavoured to fulfil the purpose in view. Judged by English standards, the regulations compare favourably with the constitution of other orders in the Roman Church for liberality, freedom, and breadth of thought and design, while their general fitness to communities of women labouring in the same field of usefulness, though under very diverse circumstances, is widely admitted.

S. Vincent de Paul died in 1660, receiving canonisation in 1737. Although his chief monument, and one likely to prove "more enduring than brass," is the organisation which has been described, his life was full of other interests. He paid great attention to criminals, especially to those condemned to the galleys. He founded hospitals for old men and women, and for deserted children. He attended Louis XIII during his last illness, and he took a prominent part in the controversy between the Jesuits and the Jansenists, employing his pen on behalf of the disciples of Ignatius Loyola. From first to last the noble spirit of S. Vincent, his self-devotion, his zeal, his piety, inspired the community of which he was the founder, and, unlike many of the older orders, the congregation of the Sisters of Charity, though not without their trials and calamities from the hostility of the world, have no period of backsliding and laxity to remember and mourn over.

